# Expression and Functions of the CB_2_ Receptor in Human Leukocytes

**DOI:** 10.3389/fphar.2022.826400

**Published:** 2022-02-22

**Authors:** Mélissa Simard, Volatiana Rakotoarivelo, Vincenzo Di Marzo, Nicolas Flamand

**Affiliations:** ^1^ Centre de Recherche de l’Institut Universitaire de Cardiologie et de Pneumologie de Québec, Département of Médecine, Faculté de Médecine, Université Laval, Québec City, QC, Canada; ^2^ Canada Excellence Research Chair on the Microbiome-Endocannabinoidome Axis in Metabolic Health (CERC-MEND), Université Laval, Québec City, QC, Canada; ^3^ Endocannabinoid Research Group, Institute of Biomolecular Chemistry, Consiglio Nazionale Delle Ricerche (CNR), Pozzuoli, Italy; ^4^ Institut sur la Nutrition et les Aliments Fonctionnels, Centre NUTRISS, École de Nutrition, Faculté des Sciences de L’agriculture et de L’alimentation, Université Laval, Québec City, QC, Canada; ^5^ Joint International Unit Between the Consiglio Nazionale Delle Ricerche (Italy) and Université Laval (Canada) on Chemical and Biomolecular Research on the Microbiome and Its Impact on Metabolic Health and Nutrition (UMI-MicroMeNu), Naples, Italy

**Keywords:** CB2 receptor, eosinophil, neutrophil, monocyte, lymphocyte, inflammation, asthma, allergy

## Abstract

The cannabinoid CB_2_ receptor was cloned from the promyeloid cell line HL-60 and is notably expressed in most, if not all leukocyte types. This relatively restricted localization, combined to the absence of psychotropic effects following its activation, make it an attractive drug target for inflammatory and autoimmune diseases. Therefore, there has been an increasing interest in the past decades to identify precisely which immune cells express the CB_2_ receptor and what are the consequences of such activation. Herein, we provide new data on the expression of both CB_1_ and CB_2_ receptors by human blood leukocytes and discuss the impact of CB_2_ receptor activation in human leukocytes. While the expression of the CB_2_ mRNA can be detected in eosinophils, neutrophils, monocytes, B and T lymphocytes, this receptor is most abundant in human eosinophils and B lymphocytes. We also review the evidence obtained from primary human leukocytes and immortalized cell lines regarding the regulation of their functions by the CB_2_ receptor, which underscore the urgent need to deepen our understanding of the CB_2_ receptor as an immunoregulator in humans.

## Introduction

The cannabinoid receptors 1 and 2 (CB_1_ and CB_2_) are two G protein-coupled receptors that function through binding a vast array of ligands including phytocannabinoids and endocannabinoids (Di Marzo et al., 1998; Turcotte et al., 2015). The CB_1_ receptor, highly expressed in the brain, was the first cannabinoid receptor identified through its responsiveness to Δ^9^-tetrahydrocannabinol (Δ^9^-THC) and cloned (Devane et al., 1988; Matsuda et al., 1990). Its activation induces psychotropic effects and its involvement shown in, among others, motor function, cognition and memory (Howlett and Abood 2017). It is also widely recognized as worsening obesity and related diseases (Di Marzo 2018). The CB_2_ receptor was later cloned from HL-60 cells and identified on its 44% aminoacid homology with the CB_1_, as well as its similar binding profile to the endocannabinoid *N*-arachidonoyl-ethanolamine (AEA) and Δ^9^-THC (Munro et al., 1993). Soon after, Galiègue et al. documented that it was expressed by human leukocytes (Galiegue et al., 1995). This consolidated the concept that the CB_2_ is the peripheral cannabinoid receptor and, for many, the inflammatory cannabinoid receptor. In fact, the CB_2_ receptor has been found in all leukocyte populations tested so far [see (Turcotte et al., 2016) for a review]. However, CB_2_ receptor expression is not restricted to leukocytes. It has notably been found in resident immune brain cells (microglia), the kidney, spleen, tonsil, thymus, lung epithelial cells and testes (Sanchez et al., 2001; Brown et al., 2002; Van Sickle et al., 2005; Ellert-Miklaszewska et al., 2007; Zhou et al., 2018; Cakir et al., 2019; Fantauzzi et al., 2020).

## Expression of the CB_1_ and CB_2_ Receptors by Human Blood Leukocytes

Galiègue et al. paved the way to our understanding of CB_2_ expression by human leukocytes by showing its mRNA was expressed in human leukocytes, with the following order of relative abundance: tonsillar B cells > natural killer cells > monocytes ∼ granulocytes > T4 lymphocytes > T8 lymphocytes (Galiegue et al., 1995). While very informative and useful, the data from Galiègue et al. did not include eosinophils while including tissue instead of blood B lymphocytes. This was somewhat pointed out in following studies (Turcotte et al., 2016), as it might have led to some inconsistencies. For example, while some documented the expression of the CB_2_ receptor in human granulocytes (neutrophils and contaminating eosinophils) (Galiegue et al., 1995; Kurihara et al., 2006), others did not (Oka et al., 2004; Graham et al., 2010). This raised the possibility that contaminating cells might have been responsible for the previously documented CB_2_ signal in neutrophils, and possibly other cell types. Noteworthy, it was later reported that eosinophil-depleted neutrophils weakly expressed the CB_2_ receptor mRNA, while eosinophils (the main neutrophil suspension contaminant) expressed it at high levels, raising the strong possibility that discrepancies regarding CB_2_ expression in neutrophils could be the result of contaminating eosinophils in granulocyte preparations (Chouinard et al., 2013). CB_2_ expression was also reported in human eosinophils in other studies (Frei et al., 2016; Larose et al., 2017; Freundt-Revilla et al., 2018; Dothel et al., 2019).

**FIGURE 1:**
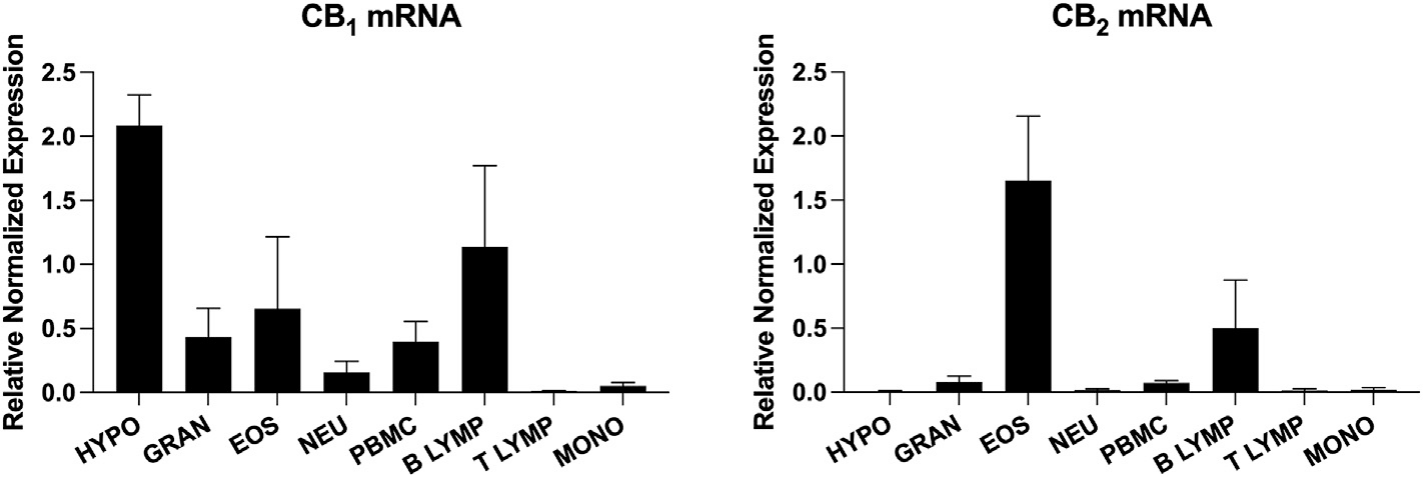
Expression of the CB_1_ and CB_2_ receptors mRNA in human leukocytes isolated from the blood. Human venous blood was collected from healthy volunteers with the informed consent of all participants in blood collection tubes containing K_3_EDTA as anticoagulant. Granulocytes (GRAN), eosinophils (EOS) and neutrophils (NEU) were isolated as in Chouinard et al. (2013). PBMCs were obtained from the PBMC layer and taken as is or otherwise processed for monocyte (MONO), B and T lymphocytes (LYMP) isolation using the EasySep™ monocyte isolation kit, CD19 positive Selection Kit II and CD3 positive selection Kit II respectively, as per the manufacturer’s protocol. Purity of the different isolated leukocytes was always >97% with the exception of B Lymphocytes (90%) with MONO being the main contaminant. Hypothalamus (HYPO) samples were obtained from the Douglas-Bell Canada Brain Bank (McGill University, Montréal, Canada). mRNA was next isolated from the different preparations with TRIzol as per the manufacturer’s protocol. 500 ng of total RNA was reverse transcribed using a High-Capacity cDNA Reverse Transcription Kit (Applied Biosystems, CA, USA) as recommended. qPCR analyses were finally performed on a CFX Connect Real-Time PCR System, using the following primers (forward - reverse): GAPDH (5′-ACA​TCG​CTC​AGA​CAC​CAT​G-3′–5′-TGT​AGT​TGA​GGT​CAA​TGA​AGG​G-3′) 18S (5′-CGC​ACG​GCC​GGT​ACA​GTG​AA-3′–5′-GGG​AGA​GGA​GCG​AGC​GAC​CA-3′) CB_1_ (5′-TTC​CCT​CTT​GTG​AAG​GCA​CTG-3′–5′-TCT​TGA​CCG​TGC​TCT​TGA​TGC-3′) and CB_2_ (5′-CAA​GGC​TGT​CTT​CCT​GCT​GA-3′–5′-CGG​GTG​AGC​AGA​GCT​TTG​TA-3′). Data represent the mean (±SEM) of 4–6 donors and was obtained using the CFX Maestro Software (Bio-Rad).

In an attempt to better define CB_2_ expression in human blood leukocytes, we revisited its expression by qPCR using mRNA from leukocytes that were isolated from the blood of healthy volunteers. CB_1_ receptor expression was assessed in parallel. Hypothalamus samples were utilized as positive controls for the CB_1_ receptor. In our hands, all tested leukocytes expressed the CB_1_ receptor mRNA although to a lesser extent than hypothalamus samples (Figure 1A). In contrast, while we detected the expression of the CB_2_ receptor mRNA in all leukocyte and hypothalamus samples, human eosinophils and B lymphocytes displayed the strongest signals (Figure 1B). Thus, these cell types are likely the origin of CB_2_ expression found in mixed populations such as granulocytes (neutrophils and eosinophils, often abbreviated as PMN) and PBMCs (monocytes, B and T lymphocytes). This underlines the importance of separating granulocytes and PBMCs when studying the CB_2_ receptor. The small, but detectable levels of CB_2_ receptor mRNA in hypothalamus samples are consistent with other studies reporting its expression in this tissue (Sanchez et al., 2001; Van Sickle et al., 2005; Ellert-Miklaszewska et al., 2007).

## Factors Influencing CB_2_ Receptor Expression in Human Leukocytes

Some factors were documented as influencing CB_2_ receptor expression in human leukocytes. CB_2_ expression can increase during inflammation as it is the case in eosinophils from symptomatic allergic donors compared to healthy controls (Frei et al., 2016; Larose et al., 2017), in monocytes of patients after ischemic stroke (Greco et al., 2021), in myeloid and plasmacytoid dendritic cells of patients with multiple sclerosis (Chiurchiu et al., 2013; Sanchez Lopez et al., 2015) and in T lymphocytes of Non-Hodgkin’s lymphomas (Rayman et al., 2007; Robinson et al., 2013). On the other hand, LPS decreased CB_2_ receptor expression in isolated dendritic cells and B lymphocytes (Lee et al., 2001; Do et al., 2004). Finally, the CB_2_ receptor was not detected in resting macrophages, was present at high levels in responsive and primed cells and was greatly diminished in fully activated cells (Cabral 2010). The latter observation suggests that the CB_2_ receptor might have a time-specific function in macrophages during inflammation.

Numerous CB_2_ receptor antibodies have been developed but most (if not all) are failing to provide reliable signals in different applications (immunohistochemistry, cytofluorometry and immunoblot), while not always having been characterized with the appropriate controls (control peptide blockade, CB_2_ receptor-devoid cells, cross reactivity). Thus, until a clear consensus is achieved on which antibodies are sufficiently reliable, data on CB_2_ protein should be interpreted with caution. With that in mind, the CB_2_ receptor protein localization can vary. Indeed, Castaneda et al. reported that the CB_2_ receptor protein was found intracellularly in most leukocytes with only B lymphocytes expressing it at the extracellular membrane (Castaneda et al., 2013). CB_2_-positive B lymphocytes were mainly located in the mantle of secondary lymphoid follicles, which contain immature B lymphocytes while some positive cells also appeared in the germinal centers of secondary follicles, which contain mature B lymphocytes, suggesting an heterogeneous distribution of the receptor during B lymphocytes maturation stages (Galiegue et al., 1995). Immunohistochemical analysis using an N-terminal specific anti-CB_2_ antibody revealed high protein expression in the germinal centers of secondary follicles while a C-terminal specific anti-CB_2_ antibody (only recognizing a non-phosphorylated inactive receptor) showed positivity primary follicle, the mantle and marginal zones of the secondary follicles where resting cells reside (Rayman et al., 2004). Therefore, active CB_2_ seems mainly present on B lymphocytes in the germinal centers.

## Impact of CB_2_ Receptor Activation in Human Leukocytes

The early studies investigating the roles of the CB_2_ receptor, notably those involving *cnr2*-deficient mice, led to the idea that it is mainly anti-inflammatory (Turcotte et al., 2016). However, recent studies are emerging and indicate that the outcome of CB_2_ receptor signaling may differ depending on the experimental model/disease. A good example is experimental asthma. Indeed, early work indicated that the CB_2_ receptor agonist WIN 55,212-2 inhibited ovalbumin-induced plasma extravasation in guinea pig airways (Fukuda et al., 2010). In contrast, the CB_2_ receptor agonist JWH-133 aggravated ovalbumin-induced asthma in mice while having no effect in dinitrofluorobenzene-induced asthma (Bozkurt et al., 2016; Frei et al., 2016). When house dust mites were utilized as allergen, *cnr2*-deficient mice were resistant to allergic responses (Ferrini et al., 2017) while an innate lymphoid cell-2 dependent model involving IL-25, IL-33 and/or *Alternaria alternate* had lower symptoms, decreased eosinophil number, and airway resistance (Hurrell et al., 2021). In humans, CB_2_ receptor expression was increased in nasal polyps of aspirin-exacerbated disease patients (Corrado et al., 2018) while being decreased in epithelial cells of asthmatic patients (Fantauzzi et al., 2020).

While we address some leukocytes individually below, the overall impact of CB_2_ receptor activation on human leukocytes is summarized in Table 1. However, we underscore that the selectivity of the pharmacological tools targeting CB_2_ receptors (agonists, antagonists, inverse agonists) has been often questioned, as exemplified by the work of Soethoudt et al. (2017).

**TABLE 1:** CB_2_-mediated effects on human leukocytes and related human cell lines.

Leukocytes or cell lines	Agonist		Antagonist or inverse agonist	Effects	Impact on signaling	References
Eosinophils						
Blood	2-AG	1 μM (4 h)	SR144528 (1 μM)	Induce migration in presence of 1 μM NDGA (lipoxygenase inhibitor)		Oka et al. (2004)
		1 μM (1 h)	SR144528 (1 μM)	2-AG-induced migration in presence of 1 μM NDGA is attributed to chemotaxis rather than chemokinesis		Kishimoto et al. (2006)
		3 μM (2 h)	SR144528 (10 μM)	Induce migration in presence of IL-5	Inhibited by the Lyn inhibitor PP2	Larose et al. (2014)
			AM630 (10 μM)			
		250 nM (5 h)	SR144528 (1 μM)	↑ CCL24-induced shape change and migration		Frei et al. (2016)
	CP 55,940	1 μM (2 h)	-	No effect on migration		Larose et al. (2014)
	JWH-133	100–250 nM (5 h)	SR144528 (1 μM)	Induce migration	Migration inhibited by MEK1 inhibitors (U-0126, PD98,059) and the ROCK inhibitor Y-27632	Frei et al. (2016)
				↑ CCL24-induced shape change and migration	Not inhibited by pertussis toxin (PTX; Gα_i_-independant), p38 or PI3K inhibitors	
				↑ CCL24-induced CD11b upregulation	- ↑ Ca^2+^ influx	
				↑ Adhesion to ICAM-1	- Ca^2+^ influx inhibited by the PLC inhibitor U-73122 and the IP3 receptor antagonist 2-APB	
Leukemia EoL-1 cells	2-AG	1 μM (4 h)	SR144528 (1 μM)	Induce migration in presence of 1 μM NDGA	Inhibited by PTX (G_i/0_-dependant)	Oka et al. (2004)
	S-777469	100–500 nM (4 h)	-	↓ 2-AG-induced migration		Haruna et al. (2017)
B lymphocytes						
Blood	CP 55,940	1–100 nM (72 h)	SR144528 (100–300 nM)	↑ Proliferation		Carayon et al. (1998)
Tonsillar	CP 55,940	1–100 nM (72 h)	SR144528 (100–300 nM)	↑ Proliferation of both naïve and germinal centrosome B lymphocytes		Carayon et al. (1998)
	WIN 55,212–2	10 μM (4 h)	SR144528 (10 nM)	No effect		Gustafsson et al. (2006)
Raji cell line	2-AG	300 nM (4 h)	SR144528 (100 nM)	Induce moderate migration		Rayman et al. (2004)
				↑ Migration following stimulation with an anti-sCD40 antibody		
Rec-1 cell line	WIN 55,212–2	10 μM (4 h)	SR144528 (10 nM)	↑ Apoptosis (caspase-3 activity)	- Inhibited by the CB_1_ inverse agonist SR141716A and by p38 inhibitors	Gustafsson et al. (2006)
				↑ Ceramide levels (downstream of p38 activation)	- Not inhibited by c-Jun or MEK-1 inhibitors	
SKW 6.4 cell line	-		SR144528 (5–10 μM)	↓ IL-6 induced secretion of soluble IgM	- Inhibited by the CB_2_ agonist HU308	Feng et al. (2014)
			AM630 (5 μM)	- ↓ IL-6-induced p-STAT3	- Do not degrade IκBα as the NF-κB inhibitor Bay11-7085	
				- ↑ Pax5 (first) and Bcl-6 mRNA levels		
Neutrophils						
Blood	2-AG	1 μM (4 h)	SR144528 (1 μM)	No effect on migration in presence of NDGA		Oka et al. (2004)
		300 nM (20 min)	SR144528 (1 μM)	No motility or morphologic alterations		Kurihara et al. (2006)
	JWH-015	100 nM-10 μM (20 min)	SR144528 (1 μM)	No motility or morphologic alterations		Kurihara et al. (2006)
	JWH-133	1 μM (2 h)	-	No effect on neutrophil function		Zhou et al. (2020)
		100 nM (5 h)	SR144528 (1 μM)	No effect on IL-8-induced migration		Frei et al. (2016)
		100 nM-1 μM (30 min)	AM630 (500 nM)	↓ LPS-induced VEGF-A		Braile et al. (2021)
				↓ LPS-induced endothelial permeability		
T lymphocytes						
Blood	AEA	0.5–5 μM (6 h)	SR144528 (1 μM)	↓ Proliferation		Cencioni et al. (2010)
				↓ IL-2, TNF-α and IFN-γ		
				↓ IL-17		
	JWH-015	20 μM (1 h)	AM630 (500 nM)	↓ CXCL12-induced chemotaxis		Ghosh et al. (2006)
		250 nM (2 h)	AM630 (500 nM)	↓ Proliferation	↓ p-ERK1/2	Borner et al. (2009)
				↓ IL-2		
		1 μM (6 h)	SR144528 (1 μM)	↓ Proliferation		Cencioni et al. (2010)
				↓ IL-2, TNF-α and IFN-γ		
				↓ IL-17		
		1 μM (1–30 min)	AM630 (1 μM)	↓ HIV-1 infection in primary CD4 T cells		Costantino et al. (2012)
	JWH-133	0.001–10 μM (30 min)	-	↓ CXCL12-induced chemotaxis	↑ p-ERK1/2	Coopman et al. (2007)
		100 nM-1 μM (1–30 min)	AM630 (1 μM)	↓ HIV-1 infection in primary CD4 T cells	↓ p-ERK1/2 and p-Akt	Costantino et al. (2012)
				↓ Activation of CXCR4 by SDF-1α		
				↓ Levels of F-actin		
	Δ^9^-THC	5 μg/ml (18 h)	SR144528 (1 μM)	↓ Percentage of T lymphocytes expressing IFN-γ		Yuan et al. (2002)
				↓ IFN-γ intracellular level detected per cell		
				↑ IL-4 and IL-5		
Jurkat cells	GW 405833	10–40 μM (3–24 h)	AM630 (1 μg/ml)	↓ Cell viability		Huang et al. (2019)
				↑ Cell apoptosis (annexin V)		
	JWH-015	20 μM (1 h)	AM630 (500 nM)	↓ CXCL12-induced chemotaxis	↑ CXCL12-induced p-ERK1/2	Ghosh et al. (2006)
				↓ Transendothelial migration	Migration not inhibited by the MEK-1 inhibitor PD 98,059	
				↓ PMA-induced MMP9		
		250 nM (2 h)	AM630 (500 nM)	↓ anti-CD3/anti-CD28-induced IL-2 production	- ↓ p-ERK1/2	Borner et al. (2009)
					- ↑ p-Lck	
					- ↓ cAMP levels	
					- Increased cAMP levels were inhibited by PTX	
	LV50	10 μM (4–72 h)	SR144528 (1 μM)	↓ T cell proliferation		Capozzi et al. (2018)
				↑ Apoptosis		
	Δ^9^-THC	1–5 μM (1–2 h)	SR144528 (2 μM)	↓ Cell viability		Herrera et al. (2006)
				↑ Apoptosis (Annexin 5)		
				↑ Ceramide levels		
				Activation of caspase 8 at a post-mitochondrial level		
Monocytes						
Blood	2-AG	10 nM–10 μM (4 h)	SR144528 (1 μM)	↑ Migration (chemotaxis toward 2-AG)		Kishimoto et al. (2003)
	(E)-β-caryophyllene	500 nM (18 h)	AM630 (5 μM)	↓ LPS-induced IL-1β and TNFα	↓ LPS-induced p-ERK1/2 and p-JNK1/2	Gertsch et al. (2008)
	JWH-015	5–20 μM (60 min)	SR144528 (1 μM)	↓ CCL2- and CCL3-induced migration	- Inhibited by PI3K and the MEK-1 inhibitors	Montecucco et al. (2008)
				↓ CCR2 and CCR1 mRNA expression	- Not inhibited by the p38 inhibitor SB-203580	
				↓ IFNγ-induced ICAM-1 induction		
		1–10 μM (20 min)	-	↓ IL-1β		Rizzo et al. (2019)
	JWH-133	1 μM (18 h)	SR144528 (1 μM)	-	↑ p-ERK1/2	Gertsch et al. (2008)
		0.1–10 μM (days 4, 7 and 10)	-	↓ HIV-1 viral infection during differentiation in monocyte derived macrophages		Williams et al. (2014)
U937 cells	2-AG	1 μM (5 min)	SR144528 (3 μM)	↑ Adhesion to fibronectin		Gokoh et al. (2005a)
	CP 55,940	1 nM–1 μM (2 h)	SR144528 (1 μM)	↓ HIV-1 transactivating protein-enhanced adhesion of cells to extracellular matrix protein, such as collagen IV and laminin		Raborn et al. (2014)
	WIN 55,212–2	1–10 μM (2 h)	AM630 (1 μM)	↓ Adhesion to HUVECs		Zhao et al. (2010)
Mast cells						
Endometrial	JWH-015	10^−8^–10^−6^ M (2 h)	-	↓ Calcium ionophore A23187-induced degranulation		Iuvone et al. (2008)
Macrophages						
Monocyte-derived macrophages (healthy subjects)	JWH-015	50 nM (30 min)	SR144528 (50 nM–0.1 μM)	↓ oxLDL-induced CD36		Chiurchiu et al. (2014)
				↓ oxLDL-induced TNF-α, IL-12 and IL-10		
	Lenabasum	0.1–30 μM (Day 0, 3, and 6)	-	No effect		Tarique et al. (2020)
Monocyte-derived macrophages (patients with cystic fibrosis)	Lenabasum	0.1–30 μM (Day 0, 3, and 6)	-	↓ Macrophage polarization into pro-inflammatory M1 phenotype		Tarique et al. (2020)
				↓ IL-8 and TNF-α secretion		
Lung	JWH-133	1 μM (10 min)	AM630 (0.5 μM)	↓ LPS-induced VEGF-A and VEGF-C	↑ p-ERK1/2	Staiano et al. (2016)
				↓ LPS-induced IL-6		
HL-60-derived macrophage	2-AG	1 μM (1 min)	SR144528 (1 μM)	Induce morphological changes such as the extension of pseudopods	- Inhibited by PTX (G_i/0_-dependant)	Gokoh et al. (2005b)
				↑ Actin polymerization	- Inhibited by selective chelating agent for intracellular free Ca^2+^ BAPTA-AM	
					- Inhibited by the PI3K inhibitor wortmannin -Not inhibited by the tyrosine kinase inhibitor herbimycin, the MEK-1 inhibitor PD 98,059 or the PKC inhibitor Ro-31–8220	
THP-1-derived macrophage M2	JWH-015	1–5 μM (12 h)	-	↓ Migration of A549 cells	↓ p-ERK1/2 and p-STAT3	Ravi et al. (2016)
Dendritic cells						
Myeloid	AEA	2.5 μM (4 h)	SR144528 (1 μM)	↓ R848-induced TNF-α, IL-12p40, IL-6		Chiurchiu et al. (2013)
	JWH-015	1 μM (4 h)	SR144528 (1 μM)	↓ R848-induced TNF-α, IL-12p40, IL-6		Chiurchiu et al. (2013)
Plasmacytoid (healthy subjects)	AEA	2.5 μM (4 h)	SR144528 (1 μM)	↓ R848-induced TNF-α, IFN-α		Chiurchiu et al. (2013)
	2-AG	10 μM (18 h)	SR144528 (1 μM)	↓ CpGA-induced IFNα		Rahaman et al. (2019)
				↓ TLR9 activation		
	JWH-015	1 μM (4 h)	SR144528 (1 μM)	↓ R848-induced TNF-α and IFN-α		Chiurchiu et al. (2013)
		0.01–1 μM (5 h)	-	↓ CpG-induced IFNα and TNFα	↓ p-IRF7, p-TBK1, p-NF-κB and p-IKKγ	Henriquez et al. (2019)
	JWH-133	0.001–0.1 μM (5 h)	-	↓ CpG-induced IFNα and TNFα	↓ p-IRF7, p-TBK1, p-NF-κB and p-IKKγ	Henriquez et al. (2019)
Plasmacytoid (patient with multiple sclerosis)	AEA	2.5 μM (4 h)	SR144528 (1 μM)	No effect		Chiurchiu et al. (2013)
	JWH-015	1 μM (4 h)	SR144528 (1 μM)	No effect		Chiurchiu et al. (2013)

### Human Eosinophils

Eosinophils participate in innate immunity against parasites and in the development/persistence of diverse inflammatory responses, notably allergies and asthma. Studies involving human eosinophils and CB receptors are scarce. Their treatment with either the endocannabinoid 2-AG and/or CB_2_ receptor agonists stimulated their migration or potentiated their migration toward other chemoattractants (Oka et al., 2004; Kishimoto et al., 2006; Larose et al., 2014; Frei et al., 2016). Importantly, these effects were prevented by the CB_2_ receptor antagonists AM630 and/or SR144528. Consistent with a CB_2_-mediated increased in eosinophil migration, cannabis use has been linked to some cases of acute eosinophilic pneumonia, although no demonstration has proven that this involved the CB_2_ receptor (Sauvaget et al., 2010; Liebling and Siu 2013; Natarajan et al., 2013; Ocal et al., 2016; Mull et al., 2020). Interestingly, while JWH-133 led to a moderate chemotactic response in human eosinophils, it had no effect on mouse eosinophils (Frei et al., 2016). Altogether, the current data support that the CB_2_ receptor stimulates eosinophil migration. This could eventually lead to increased parasitic defenses but also to a worsening of eosinophils-related inflammatory diseases.

### Human B Lymphocytes

B lymphocytes maturation and differentiation are complex processes. Following their activation, naïve cells (spleen marginal zone) proliferate and differentiate into short-lived plasma cells, while cells from the follicles undergo massive proliferation and form germinal centers, where long-lived plasma and memory cells are formed (Basu et al., 2013). Very little is known about the role of the CB_2_ receptor in human B lymphocytes but their treatment with CP 55,940 increased their proliferation, a phenomenon blocked by SR144528 (Carayon et al., 1998). In mice, activation of the CB_2_ receptor has been associated with B lymphocyte differentiation, migration, proliferation and antibody class switching (Jorda et al., 2002; Tanikawa et al., 2007; Agudelo et al., 2008), suggesting the receptor is part of the B lymphocytes immune programing, playing an important role in B lymphocyte repertoire formation (Pereira et al., 2009).

### Human Neutrophils

Neutrophils are first responders of the innate immune system, playing crucial roles in acute inflammatory responses and host defense. They employ several strategies to fight microbes, including the phagocytosis and killing of pathogens with the help of their granule content. Studies showing a CB_2_-receptor-mediated effect of human neutrophils were not conclusive and contaminating eosinophils in neutrophil preparations might have caused a red herring situation, eosinophils being responsible for most of the CB_2_ receptor signal/effects (Figure 1 and *Expression of the CB*_*1*_ *and CB*_*2*_ *Receptors by Human Blood Leukocytes*). In fact, numerous studies indicated that endocannabinoids as well as selective and non-selective CB_2_ receptor agonists do not diminish human neutrophil functions (migration, superoxide generation and degranulation) *via* the CB_2_ receptor and when they display an inhibitory effect on their functional responses it is mostly related to a mechanism distinct from the CB_1_ and CB_2_ receptors (Deusch et al., 2003; Kraft et al., 2004; Oka et al., 2004; McHugh et al., 2008; Chouinard et al., 2011; Montecucco et al., 2012; Zhou et al., 2020), which is consistent with their lack/very low expression of the CB_2_ receptor. In contrast, JWH-133 inhibited the release of VEGF-A but not CXCL8 from LPS-stimulated human neutrophils, a phenomenon prevented by the CB_2_ receptor antagonist AM630 (Braile et al., 2021).

• *In vivo* studies indicated that mouse neutrophils are more responsive to CB_2_ receptor activation than human neutrophils. As such, *Cnr2* ^−/−^ mice models reported increased neutrophil numbers at inflammatory sites (Alferink et al., 2016; Kapellos et al., 2017; Kapellos et al., 2019). Accordingly, CB_2_ activation by selective agonists suppressed neutrophil recruitment to the inflammation site (Horvath et al., 2012; Andrade-Silva et al., 2016; Wang et al., 2016; Parlar et al., 2018; Kapellos et al., 2019). However, it is not clear whether the reported evidence is a matter of mouse neutrophil responsiveness or of indirect CB_2_-dependent effects mediated by other cells (Kraft and Kress 2005). At this point, we cannot exclude that a CB_2_-dependent mechanism prevents neutrophil recruitment into by impairing their transmigration into the tissues and by affecting other cells (e.g., endothelial cells) as proposed earlier (Nilsson et al., 2006).

### Human T Lymphocytes

Cytotoxic CD8 T lymphocytes are responsible for the elimination of invading/dysfunctional cells while CD4 T lymphocytes produce a myriad of inflammatory mediators and are referred to as helper lymphocytes (Th). Although CB_2_ receptor expression was barely detected in circulating T lymphocytes (Figure 1), several studies reported that CB_2_ receptor expression is increased in activated T lymphocytes and that its activation decreases their proliferation (Borner et al., 2009; Cencioni et al., 2010; Capozzi et al., 2018). This is accompanied with decreased IL-2 production and increased apoptosis (Herrera et al., 2006; Borner et al., 2009; Cencioni et al., 2010; Capozzi et al., 2018; Huang et al., 2019). Interestingly, CB_2_ receptor activation seems to exert divergent effects depending on the T lymphocyte subtype with the tendency to decrease human Th1 and Th17 functions, while promoting those of Th2. For instance, Δ^9^-THC decreased in a CB_2_-dependant manner the percentage of human T lymphocytes expressing IFN-γ, and intracellular levels of IFN-γ per cells (Th1), while increasing levels of IL-4 and IL-5 (Th2) (Yuan et al., 2002). Accordingly, a decrease in IL-17 levels was found in JWH-015-treated T lymphocytes (Cencioni et al., 2010). Finally, the CB_2_ agonist Lenabasum reduced TNF-α in both CD8 and CD4 T lymphocytes (Th1). The treatment also decreased IL-17 levels (Th17) as well as Th1 and Th17 respective signature transcription factors T-bet and RORγt (Tiberi et al., 2021).

### Human Monocytes

Blood monocytes migrate into tissues where they differentiate into macrophages or convert into non-classical monocytes (Guilliams et al., 2018). 2-AG is a CB_2_-dependant human monocyte chemoattractant (Kishimoto et al., 2003) and induces the adhesion of human monocytic U937 cells to fibronectin (Gokoh et al., 2005a). However, JWH-015 decreased the CCL2-and CCL3-induced migration of human monocytes by decreasing their receptors’ expression (Montecucco et al., 2008). JWH-015 also reduces human monocyte differentiation and U937 cells adhesion to extracellular matrix proteins, both induced by HIV-1 (Raborn et al., 2014; Williams et al., 2014). Finally, CB_2_ receptor engagement in human monocytes was shown to decrease the LPS-induced IL-1β and IL-6 production (Gu et al., 2019; Rizzo et al., 2019).

### Human Macrophages

Macrophages are resident cells that are remarkably versatile, exerting important roles in development, homeostasis, tissue repair and immunity. The endocannabinoid 2-AG was found to induce shape changes of HL-60-derived macrophages in a CB_2_-depandent manner (Gokoh et al., 2005b). Additionally, CB_2_ receptor activation with JWH-015 or JWH-133 decreased the LPS-induced VEGF-A, VEGF-C IL-6 release, as well as the oxLDL-induced release of TNF-α, IL-12 and IL-10 (Chiurchiu et al., 2014; Staiano et al., 2016). In mice, the CB_2_ receptor was shown to switch the polarization of M1 macrophage into M2 macrophage (Duerr et al., 2014; Denaes et al., 2016; Du et al., 2018). Such a phenomenon has been partially observed in humans by Tarique et al. who showed that Lenabasum decreased the polarization (M1) of monocyte-derived macrophage obtained from cystic fibrosis patients (Tarique et al., 2020).

### Human Mast Cells

Mast cells are strategically located at the interface with the external environment, acting as key initiators of local inflammatory responses (Elieh Ali Komi et al., 2020). The first evidence that they could be regulated by the CB_2_ receptor came from the rat basophilic leukemia cell line (RBL-2H3) expressing the CB_2_ receptor (Facci et al., 1995). However, while the authors showed that *N*-palmitoyl-ethanolamine (PEA) inhibited serotonin release AEA did not. However, PEA interacts with PPARα (Lo Verme et al., 2005) and its initial effects are likely linked to PPARα. In humans, the treatment of isolated mast cells with JWH-015 decreased their degranulation *in vitro* (Iuvone et al., 2008).

### Human Dendritic Cells

Dendritic cells are sentinels of the immune system bridging the innate and adaptive immunity by ingesting pathogens and transporting antigens to lymphoid tissues. Stimulation of CB_2_ receptor with CB_2_ receptor agonists reduced their cytokine production. Indeed, AEA and JWH-015 decreased R848-induced levels of TNF-α, IL-12p40 and IL-6 by myeloid dendritic cells while AEA, 2-AG, JWH-015 and JWH-133 decreased levels of R848-and/or CpG-induced IFN-α by plasmacytoid dendritic cells by a mechanisms involving NF-κB and IKKγ signalization (Chiurchiu et al., 2013; Henriquez et al., 2019; Rahaman et al., 2019).

## Conclusion

It is becoming clear that the CB_2_ receptor plays important roles in the regulation of several inflammatory processes. However, while the first studies investigating the role of this receptor in mice led to the concept that its function was mainly anti-inflammatory, new evidence is challenging this concept, notably in allergic diseases, which usually involve cells such as eosinophils and B lymphocytes, whose functional responses to CB_2_ receptor activation simulates them, in human-based studies. Moreover, the scarcity of human studies investigating the CB_2_ receptor makes our understanding of the latter difficult at this point and underscores the urgency of performing additional work involving human samples/cells to deepen our understanding of CB_2_-receptor-driven inflammatory responses and establish to what extent we can translate findings from experimental models to the clinic. It is thus urgent to further characterize the functions of the CB_2_ receptor in human leukocytes and inflammatory diseases.

## Data Availability

The original contributions presented in the study are included in the article, further inquiries can be directed to the corresponding author.
